# The Bacillus of Diphtheria

**Published:** 1890-07-05

**Authors:** 


### THE BACILLUS OF DIPHTHERIA.

Löffler, in an exhaustive review of this question, comes to the conclusion that recent observations in the most different and mutually independent quarters confirm the specific character of that bacillus which he, so long since as 1884, identified among many other forms present in diphtheria as the pathogenic one, and which, he insists, has nothing whatever to do with the so-called diphtheritic throat of scarlatina. But one observation of his own is especially interesting, viz., that among the rabbits inoculated with it, two which did not as usual die suffered some time later from characteristic paralyses, from which they recovered in due time. Since the bacilli were found nowhere except at the seat of inoculation, it would appear that these remote effects were brought about by some poison formed by the bacillus in the animal fluids.

